# Structure and thermal behavior of biobased vitrimer of lactic acid and epoxidized canola oil

**DOI:** 10.1039/d3ra06272d

**Published:** 2023-11-16

**Authors:** João Gabriel P. Rodrigues, Santiago Arias, Jose G. A. Pacheco, Marcos Lopes Dias

**Affiliations:** a Instituto de Macromoléculas Professora Eloisa Mano, Universidade Federal do Rio de Janeiro Brazil jgprodrigues@ima.ufrj.br; b Chemistry Institute, Federal University of Pernambuco Brazil

## Abstract

Biobased vitrimers were obtained from epoxidized canola oil (ECO) and lactic acid (LA) using zinc acetate (ZnAc) and ZnAl-layered double hydroxide (ZnAl) in the proportions of 1 and 2 wt% as transesterification catalysts. Reactions containing ECO and LA showed an average enthalpy of cure of approximately 85 mJ mg^−1^ and materials cured in the presence of the catalysts showed lower enthalpies of cure and decrease in the material gel content. ECO-LA reaction generated materials with rubber-like properties with *T*_g_ ranging from −15 °C to −23 °C, where the material without a catalyst showed the higher *T*_g_ value. The presence of catalysts gave the material vitrimer properties, with the softening point associated with transesterification reactions and topology freezing temperature transition at temperatures (*T*_v_) between 195-235 °C.

## Abstract 2

Biobased vitrimers were obtained from epoxidized canola oil (ECO) and lactic acid (LA) using zinc acetate (ZnAc) and ZnAl-layered double hydroxide (ZnAl) in the proportions of 1 and 2 wt% as transesterification catalysts.

## Graphical abstract

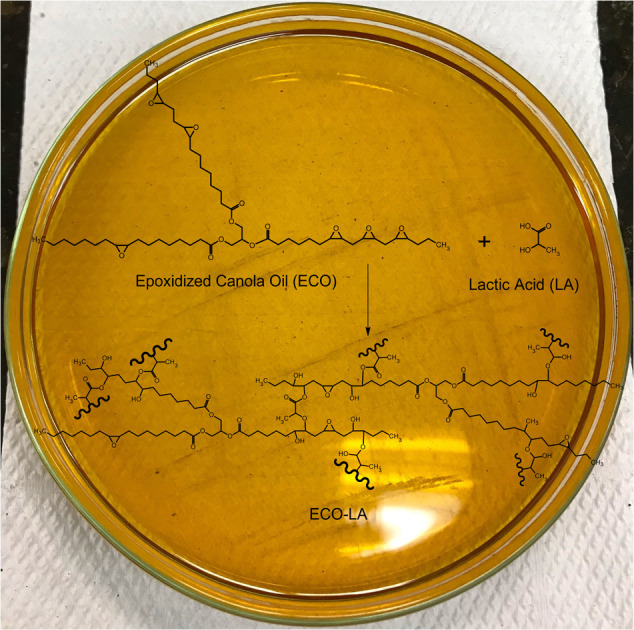

## 1 Introduction

Epoxy thermosets are highly attractive because of important material characteristics including thermal stability, resistance to chemical solvents and good mechanical properties. However, due to their permanent covalent crosslinked networks, they are not capable of being recycled, remodeled, or reused, resulting in disposal after suffering damage or failure, which is a major disadvantage for this type of material.^1–3^ In this context, vitrimers have emerged as thermoset materials capable of being re-processed and, in many cases, presenting self-healing and shape memory properties.^4^

Vitrimers represent a new group of polymers that can offer a combination of the advantages existing in thermoplastics and thermosets. These thermosetting polymers are malleable at high temperatures and become solid when cooled. This property of vitrimers is associated with the existence of Associative Dynamic Covalent Adaptive Networks in the structure of these thermosets.^5–7^ Several vitrimers obtained from different types of dynamic covalent bonds have been reported in the literature. The chemical bonds used to achieve this dynamism include olefin metathesis,^8^ disulfide exchange,^9,10^ boronic ester exchange,^11^ transimination reaction,^12^ and transesterication reaction.^13,14^

Vitrimers with dynamic transesterification reaction (DTER) are the most extensively studied by researchers because they can be applied in several types of commercial epoxy resins. DTERs occur between ester and hydroxyl bonds at elevated temperatures, altering the crosslinked structure of the material and resulting in viscous deformation of the cured material.^15^

The existing variety of epoxy monomers, polycarboxylic acids and anhydrides has enabled the formation of vitrimers with a wide range of thermal and mechanical properties. Transesterification reactions in vitrimers can occur in the absence of catalysts, but normally these reactions are typically accelerated by a variety of specific catalysts, such as Lewis and Brønsted, zinc salts, triphenylphosphine and tertiary amines.^16^

Due to the growing concern with issues related to the environment, biobased transesterificaiton vitrimers obtained from aromatic, cycloaliphatic and aliphatic chemical structures found in nature have emerged as an environmentally friendly option.^17^ Epoxidized vegetable oils have emerged as a promising option in the synthesis of vitrimers. The use of epoxidized vegetable oils can be seen as an interesting strategy for the synthesis of ecologically correct vitrimers.^18^ These oils may have several epoxy groups with the potential to undergo polymerization reaction *via* ring opening in the presence of chemical compounds containing active hydrogens, which function as curing agents, producing a crosslinked network.^19^ An abundant carboxylic acid from renewable sources that can act as a curing agent for epoxidized oils are citric, malic and lactic acids.^20,21^

Canola oil is the third most produced vegetable oil on a global scale, behind only palm oil and soybean oil. However, canola seed can produce more oil, surpassing olives by 1.5 times and soybeans by 2 times in terms of oil content.^22–24^ In its chemical structure, canola oil has a low content of saturated fatty acids and high levels of unsaturated fatty acids, such as oleic acid (C18:1(44–75%)), linoleic acid (C18:2(18–22%)) and linolenic acid (C18:3(9–13%)).^25,26^ Therefore, canola oil is a potential input for obtaining vitrimeric materials.

Lactic acid is an organic acid obtained by the selective oxidation of raw materials derived from biomass.^27^ Lactic acid acts as a chemical intermediate in the pharmaceutical field and acts as a raw material in the production of poly(lactic acid). This acid gives rise to completely degradable and environmentally friendly materials, which can be degraded into CO_2_ and H_2_O by microorganisms or acid action. Furthermore, lactic acid has the advantage of its easy availability and low cost.^28^

Vitrimers based on vegetable oils have specific applications due to the self-healing property. They also have applications in the polymeric coatings.^29^ Due to low toxicity, vegetable oil vitrimers have found space for biomedical applications with good biocompatibility in cell culture experiments.^30^

In this context, this work investigated the preparation and thermal behavior of vitrimers obtained from epoxidized canola oil and lactic acid as a curing agent in the presence of two different types of metallic catalysts at different concentrations as DTER promoters.

## 2 Experimental

### 2.1 Materials

**Fig. 1:**
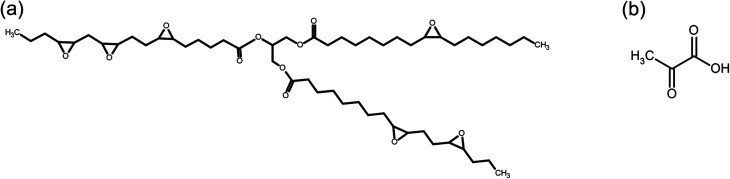
(a) Chemical structure of epoxidized canola oil and (b) chemical structure of lactic acid molecule.

The materials with vitrimeric properties were obtained from epoxidized canola oil (molar mass of 865 g mol^−1^ by GPC and epoxide equivalent weight of 229.75 g eq^−1^), synthesized according method previously reported.^31^ Racemic lactic acid (LA) (84.5–85.5%) used as a curing agent was supplied by VETEC (Brazil). The monomers used in this work can be seen in Fig. 1. Zinc acetate (ZnAc) (purity ≥ 98%) used as an homogeneous catalyst was supplied by Sigma Aldrich.

The ZnAl-layered double hydroxide (ZnAl) heterogeneous catalyst (Zn/Al molar ratio = 4.0) was synthesized through coprecipitation at low supersaturation while maintaining a controlled pH of 7.5, as previously described.^32,33^ Tetrahydrofuran (THF) (purity 99%) purchased by B'Herzog, (Germany) and chloroform (purity 99.8%) provided by Neon (Brazil) was used as received.

### 2.2 Synthesis of epoxidized canola oil vitrimers

To produce the vitrimers, epoxidized canola oil and dried lactic acid were mixed in an oil : acid molar ratio = 2 : 1. Two concentrations of the metallic catalysts (1 and 2 wt%) were used. Thus, in a glass flask, the metal catalysts were weighed and solubilized/dispersed, ZnAc in distilled water and ZnAl in THF at room temperature. After solubilization/dispersion of the catalysts, lactic acid was added and mixed at room temperature. Then, the epoxidized oil was added and mixed with the lactic acid/catalyst mixture. These pre-mixes were poured into a silicone mold and preheated in an oven at 130 °C for isothermal curing for a period of 504 hours. Table 1 presents the names of all materials formulations used in this study.

**Table 1:** Sample name and composition for all vitrimers formulations

Sample name	ECO/LA (molar ratio)	Amount of ZnAc (wt%)	Amount of ZnAl (wt%)
ECO-LA	2 : 1	0	0
ECO-LA (1%ZnAc)	2 : 1	1	0
ECO-LA (2%ZnAc)	2 : 1	2	0
ECO-LA (1%ZnAl)	2 : 1	0	1
ECO-LA (2%ZnAl)	2 : 1	0	2

### 2.3 Material characterization

Attenuated Total Reflection Fourier Transform Infrared (ATR- FTIR) spectroscopy was carried out in a Nicolet iS10 equipment (Thermofisher) using a selenide crystal, to analyze the chemical structure of the vitrimer during the curing reaction in the presence of the catalysts. Analyses were performed in a range of wavenumber from 700–4000 cm^−1^, with a resolution of 4 cm^−1^ and 32 scans.

Material soluble fraction and swelling test were performed using sample with dimensions of approximately 5 mm × 5 mm × 1 mm immersed in chloroform for a period of 72 hours at room temperature. Swelled samples were weighed and subsequently taken to an oven at a temperature of 100 °C for a period of 24 hours to obtain the dry mass. The swelling ratio and soluble fraction were calculated using eqn (1) and (2), respectively.^34^ The reported results are the average of three different measurements.1
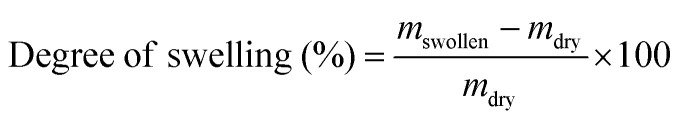
2
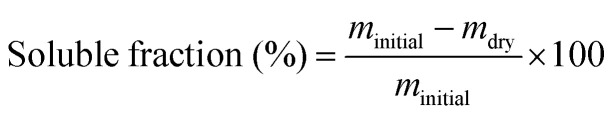


Analysis of Variance (ANOVA) was employed using *F* test to verify and determine if there is any statistically significant difference between the mean values obtained in the gel content test. A 95% confidence level was used for all tests. After verifying the existence of a statistical difference between the means of the results obtained for the two ZnAc and ZnAl concentrations, the Tukey test was used to perform multiple comparisons. Statistical analysis was performed using the OriginLab 2023b software.

The glass transition temperature (*T*_g_) of the materials and the curing behavior were determined in a Hitachi model 700 Differential Scanning Calorimeter (DSC). The curing enthalpies were calculated using the TA7000-Hitachi software. Calibration of temperature and enthalpy was performed with a high purity indium standard. For the glass transition temperature determination, approximately 10 mg of the cured materials were weighed into a closed aluminum pan and heated from 20-200 °C at a heating rate of 10 °C min^−1^, under nitrogen atmosphere at a constant flow rate of 50 ml min^−1^. For the non-isothermal kinetic study, about 10 mg of the pre-mix, with and without catalyst, were weighed into an open aluminum pan and samples and heated from 20 to 200 °C at different heating rates (4, 6, 8 and 10 °◦C min^−1^).

Thermal stability and kinetics of degradation investigation were carried out in a TA Instruments model Q-500 equipment by heating the samples from room temperature to 700 °C under a constant nitrogen flow of 50 ml min^−1^, at a heating rate of 10 °C min^−1^. For the study of degradation kinetics, the samples were tested from room temperature to 600 °C using 3 different heating rates (5, 10 and 15 °C min^−1^).

A Shimadzu Thermomechanical Analyzer (TMA) model TMA- 60 was employed to determine the topology freezing temperature (*T*_v_). Cylindrical samples measuring approximately 30 mm in diameter and 2 mm in height were tested. Analyses were performed with a glass probe, applying a constant load of 0.2 newton and constant nitrogen flow of 50 ml min^−1^, from room temperature to 250 °C and heating rate of 5 °C min^−1^.

## 3 Results and discussion

### 3.1 Structure of canola oil–lactic acid vitrimers

Reaction of canola epoxidized oil and acid lactic in presence of zinc acetate (ZnAc) and ZnAl-layered double hydroxide (ZnAl) or without these zinc-based compounds generated rubber-like poly-hydroxyesters solid resulted from ring-opening polymerization of epoxy groups of the epoxidized oil by lactic acid. This polymer network formation occurred even in the absence of the catalysts.

**Fig. 2:**
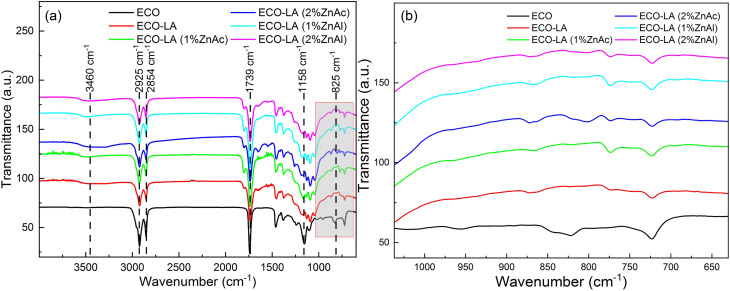
(a) FTIR spectra of ECO, ECO-LA materials without catalyst and with zinc-based catalysts at different concentrations. (b) FTIR spectra in the region of the epoxy group.

Fig. 2a shows the FTIR spectra for the epoxidized canola oil (ECO), for the polymer of epoxidized canola oil cured with lactic acid (ECO-LA) and for the vitrimers obtained with different concentrations (1 and 2 wt%) of ZnAc and ZnAl. Fig. 2b presents the epoxy group region of the FTIR spectra.

All samples show absorption bands common to vegetable oils. The spectra presented absorption bands at 2925 cm^−1^ and 2854 cm^−1^ associated with the asymmetric bending stretching of the olefinic group,^35^ a band at 1739 cm^−1^ attributed to carbonyl (C=O) stretching and at 1158 cm^−1^ associated to (C–O–C) bond stretching, both from the ester group.^36^ For ECO, the spectrum shows an absorption band at 825 cm^−1^ corresponding to the stretching of epoxy group.^37^

For all cured samples, including that cured only with LA and those cured in the presence of the two different catalysts at different concentrations was noticed the total disappearance of the absorption band related to epoxy group, suggesting that this chemical group reacted with the carboxyl and hydroxyl groups present in the LA molecule, resulting in a ring-opening polymerization reaction,^38^ (Fig. 2b). The appearance of a band at 3460 cm^−1^ was observed for the cured materials. This band is associated with the stretching of the hydroxyl group (C–OH) which is a product of the ring opening reaction. FTIR spectra also showed an increase in the intensity of the band at 1739 cm^−1^ of the ester group of the hydroxyesters framework, characteristic of vitrimers obtained from epoxy resins.^39–41^

### 3.2 Influence of catalyst on glass transition

**Fig. 3:**
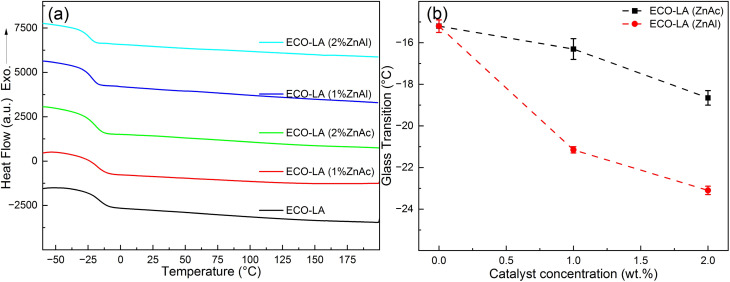
(a) DSC curves for all samples. (b) Glass transition temperature as a function of catalyst concentration.

Fig. 3a presents the DSC curves for the ECO-LA vitrimers in the presence of zinc catalysts at different concentrations. Fig. 3b shows the variation of the glass transition temperature (*T*_g_) as a function of the amount of catalyst.

It is evident that there is a reduction in *T*_g_s with the increase in the zinc-based catalysts used in the curing of the material. This behavior is observed for both types of catalyst. The material cured only with LA had a glass transition of approximately −15.2 °C, while materials cured with 1 and 2 wt% ZnAc had a *T*_g_ of −16.3 °C and −18.65 °C, respectively. For the materials obtained with 1 and 2 wt% of ZnAl, the decrease in the *T*_g_ was more pronounced, showing the transition temperatures around −21.15 °C and −23.1 °C, respectively. This reduction in *T*_g_s with increasing catalyst concentration may be associated with the flexibility of the polymer network caused by a possible reduction in the degree of crosslinking. The observed behavior of decreasing in glass transition by addition of fillers has already been reported in the literature for epoxy-based vitrimers.^42,43^ It was also related with a weak interaction between the polymer and the catalysts.^44^

#### 3.2.1 Curing kinetics analysis

**Fig. 4:**
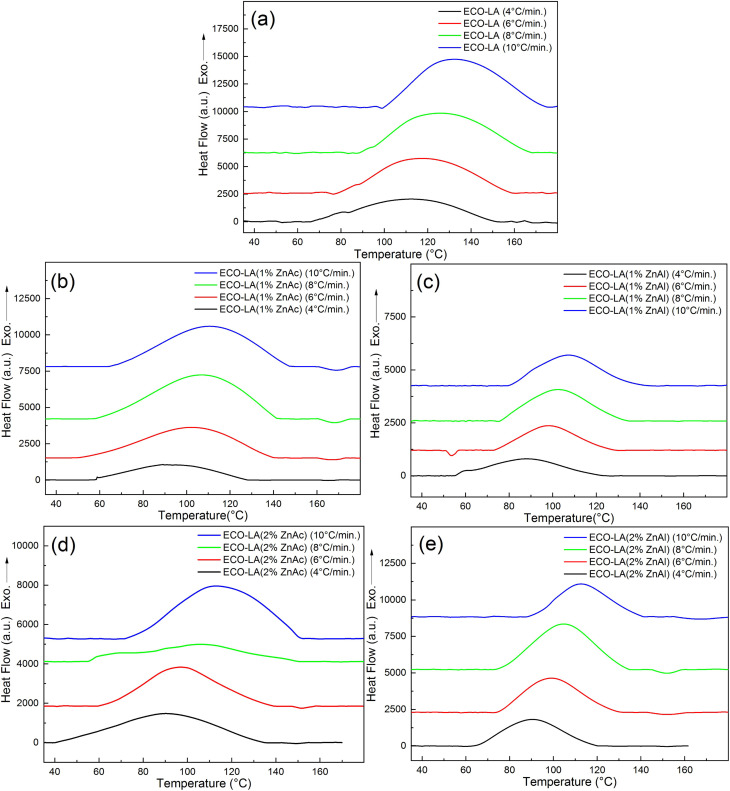
DSC curves showing exothermic curing peaks at different heating rates for (a) ECO-LA, (b) ECO-LA(1%ZnAc), (c) ECO-LA(1%ZnAl), (d) ECO-LA(2%ZnAc) and (e) ECO-LA(2%ZnAl).

To evaluate the curing process of epoxidized canola oil with lactic acid without catalysts and in the presence of ZnAc and ZnAl, reactants were mixed and subjected to non-isothermal DSC analyses at different heating rates. Fig. 4 presents the DSC curves related to the curing reaction. The curing process resulted in exothermic peaks over a temperature range of 40–180 °C.

The degree of cure (*α*) was obtained from non-isothermal DSC measurements, and it is equal to the ratio between the area under the exothermic peak at a given temperature (Δ*H*_T_) and the total heat of curing reaction (Δ*H*_∞_)^45–48^ (eqn (3)):3
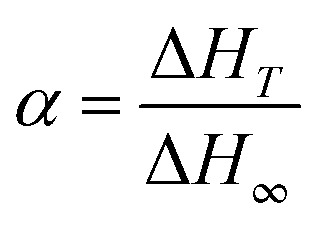


**Fig. 5:**
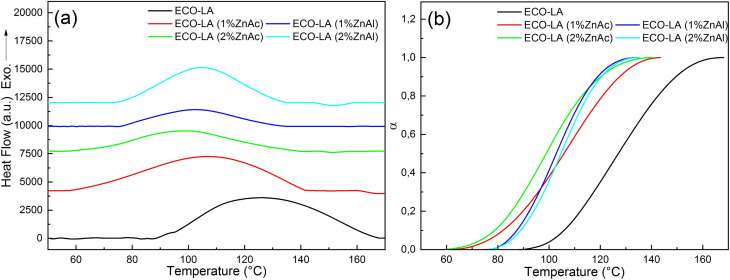
(a) DSC curves showing exothermic curing of ECO-LA vitrimers and (b) degree of cure at a heating rate of 8 °C min^−1^.

Fig. 5a shows the DSC curves for the kinetic study at a rate of 8 °C min^−1^ for materials catalyzed with ZnAc and ZnAl at different concentrations, and Fig. 5b shows the degree of cure at the same heating rate for all samples.

The dynamic thermal curing of systems containing only ECO and LA presents an exothermic peak with maximum (*T*_p_) at 125 °C. Addition of 1 and 2 wt% of ZnAc and ZnAl promotes a decrease in the temperature range of the exothermic peak and *T*_p_, and a considerable decrease in the enthalpy of cure. These values can be observed in Table 2.

**Table 2:** Kinetic data of curing of ECO-LA polymers

Sample	HR* (°C min^−1^)	Δ*H*_∞_ (mJ mg^−1^)	Mean	TP (°C)	*E*_a_ (FWO) (kJ mol^−1^)	*R* ^2^	*E*_a_ (Kissinger) (kJ mol^−1^)	*R* ^2^
ECO-LA	4	−69.6	−84.35 ± 14.88	113.19	55.79	0.96	51.80	0.95
	6	−70.8		117.39				
	8	−92		126.2				
	10	−105		132.36				
ECO-LA(1%ZnAc)	4	−60.6	−66.32 ± 7.67	89.20	44.42	0.96	43.67	0.95
	6	−61.8		102.41				
	8	−63.4		107.56				
	10	−79.5		111.08				
ECO-LA(2%ZnAc)	4	−32.1	−26.8 ± 7.67	90.6	41.38	0.97	39.68	0.96
	6	−24.1		96.82				
	8	−23		106.12				
	10	−28		113.07				
ECO-LA(1%ZnAl)	4	−71.5	−36.67 ± 20.34	88.23	52.10	0.99	48.61	0.98
	6	−25.2		98.39				
	8	−29.4		102.69				
	10	−20.6		107.44				
ECO-LA(2%ZnAl)	4	−69.8	−52.12 ± 13.76	90.70	49.22	0.99	45.53	0.99
	6	−52.8		99.00				
	8	−54.7		104.80				
	10	−31.2		111.60				

These results suggest that the addition of these zinc-based compounds acts accelerating ECO-LA reactions and that the variation in the catalyst concentration promotes significant changes in reaction kinetic in relation to the system containing only ECO and LA.^49,50^

It can also be observed that the material cured without the catalyst has a higher average value of Δ*H* in relation to materials obtained with ZnAc and ZnAl. The decrease in the enthalpy of cure is more pronounced for samples with higher concentrations of both catalysts. Decrease in the enthalpy of cure can be attributed to decrease in conversion by reducing crosslink density.^51–53^ Such variations in the crosslinking of the material can generate significant decreases in the *T*_g_ of the materials.

For kinetic studies of epoxy resins, there are several methods and models proposed to calculate some important parameters, such as the Activation Energy (*E*_a_) and the Pre-exponential Factor (*A*). Therefore, ASTM E698-18 (Flynn-Wall-Ozawa (FWO), eqn (4)) and ASTM E2890-12 (Kissinger Method, eqn (5))^54,55^ methods, were used in this work and are represented by the following equations, respectively:4
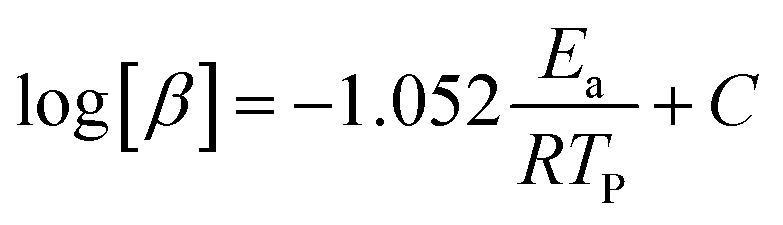
5
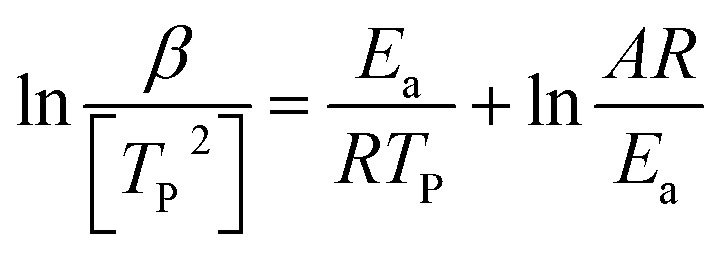


For the FWO method, a plot of log[*β*] *versus* the reciprocal of absolute temperature of the exothermic peak is performed.

The slope gives the value of *E*_a_/*R*, where *R* is the universal gas constant, *β* is the heating rate and *T*_P_ is the temperature of the exothermic peak. For the Kissinger method, a plot of 
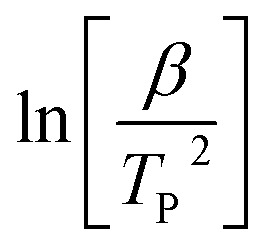
*versus* the reciprocal of the exothermic peak temperature is used, and activation energy and the pre-exponential factor are obtained through the slope and the intercept, respectively.^56–58^

**Fig. 6:**
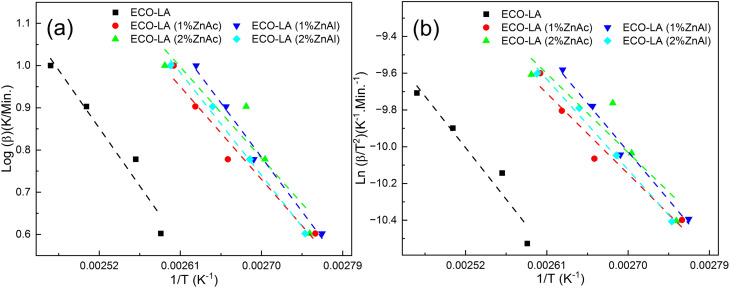
(a) Ozawa's and (b) Kissinger's plots for ECO-LA vitrimers without and with ZnAc and ZnAl at different concentrations.

**Fig. 7:**
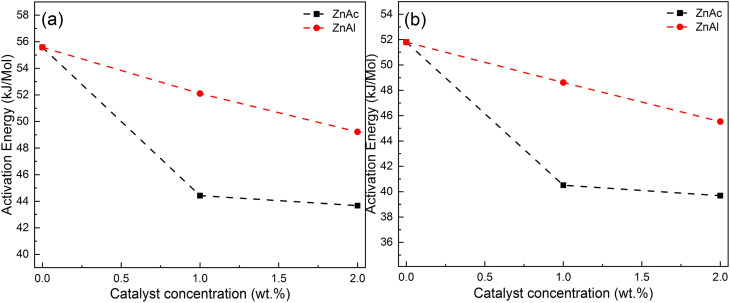
Activation energies for ECO-LA vitrimers without and with ZnAc and ZnAl at different concentrations (a) by Ozawa's and (b) Kissinger's plots.

Fig. 6a and b show the results obtained with the Ozawa and Kissinger plots for samples without catalyst, with ZnAc and ZnAl at different concentrations. Fig. 7a and b show the activation energy variation as a function of the catalyst and the mass quantity of catalyst used in the reaction calculated by both methods.

The material cured without any catalyst presents an activation energy of 55.79 kJ mol^−1^ (FWO) and 51.80 kJ mol^−1^ (Kissinger), values similar to those found in the literature. For the materials synthesized with ZnAc, the activation energy decreased 20.38% and 25.38% (Ozawa method) for materials cured with 1 and 2 wt%, respectively, in relation to the material cured without catalyst. By using the Kissinger method, the material without catalyst presented an activation energy of 51.80 kJ mol^−1^. By this method, the decrease in the activation energy was 21.79% and 28% for materials with 1 and 2 wt% ZnAc, respectively.

### 3.3 Gel content and swelling

Table 3 presents values of gel content and swelling ratio for ECO-LA materials studied in this work. A significant impact on the gel content of materials can be observed when the catalysts were used. The material synthesized without any catalyst showed an average value of 74.5% of chloroform insoluble fraction. For samples catalyzed with ZnAc, a decrease of 5% and 16.6% in the in-soluble fraction is observed for materials with 1 and 2% wt of ZnAc, respectively. This result shows that for these concentrations these catalysts can impact the density of crosslinking in the final polymer network. The same behavior was observed for the ZnAl catalysts, where the reductions in gel content were 11.7 and 19.9% for concentrations of 1 and 2 wt%, respectively. Such characteristics were confirmed through the swelling ratio. These results suggest that zinc-based fillers used in vitrimer cure decrease the density of crosslinks in the material network, corroborating the results found in kinetics curing studies and glass transition temperatures.^59^

**Table 3:** Gel content and degree of swelling for the ECO-LA materials

Material	Gel content (%)	Swelling (%)
ECO-LA	74.72 ± 0.89	548.3 ± 12.54
ECO-LA (1%ZnAc)	69.63 ± 2.87	559.7 ± 10.12
ECO-LA (2%ZnAc)	57.74 ± 1.29	1042 ± 83.06
ECO-LA (1%ZnAl)	63.27 ± 1.76	1005 ± 40.46
ECO-LA (2%ZnAl)	54.38 ± 0.66	1308 ± 58.03

Tables 4 and 5 present the ANOVA results for materials cured at different concentrations of ZnAc and ZnAl catalysts. The results for the samples containing ZnAc revealed that the calculated *F* value of 39.15 exceeded the critical *F* value (*p* value) of 3.60 × 10^−4^, indicating that the means are significantly different with 95% reliability. Materials containing the ZnAl catalyst showed a *F* value of 138.30 and a *p* value of <0.0001, revealing that for these concentration, the mean gel contents are significantly different.

**Table 4:** Analysis of variance for ECO-LA materials cured with ZnAc at different concentrations

ANOVA (ZnAc)					
Source of variation	SS	DF	MS	*F* value	*p* value
Between groups	420.12	2	210.06	39.15	3.60 × 10^−4^
Error	32.19	6	5.36		
Total	452.31	8			

**Table 5:** Analysis of variance for ECO-LA materials cured with ZnAl at different concentrations^a^

ANOVA (ZnAl)					
Source of variation	SS	DF	MS	*F* value	*P* value
Between groups	599.76	2	298.88	138.30	<00001
Error	13.01	6	2.17		
Total	612.77	8			

a HR = heating rate, SS = sum of squares, DF = degrees of freedom, MS = mean squares.

**Fig. 8:**
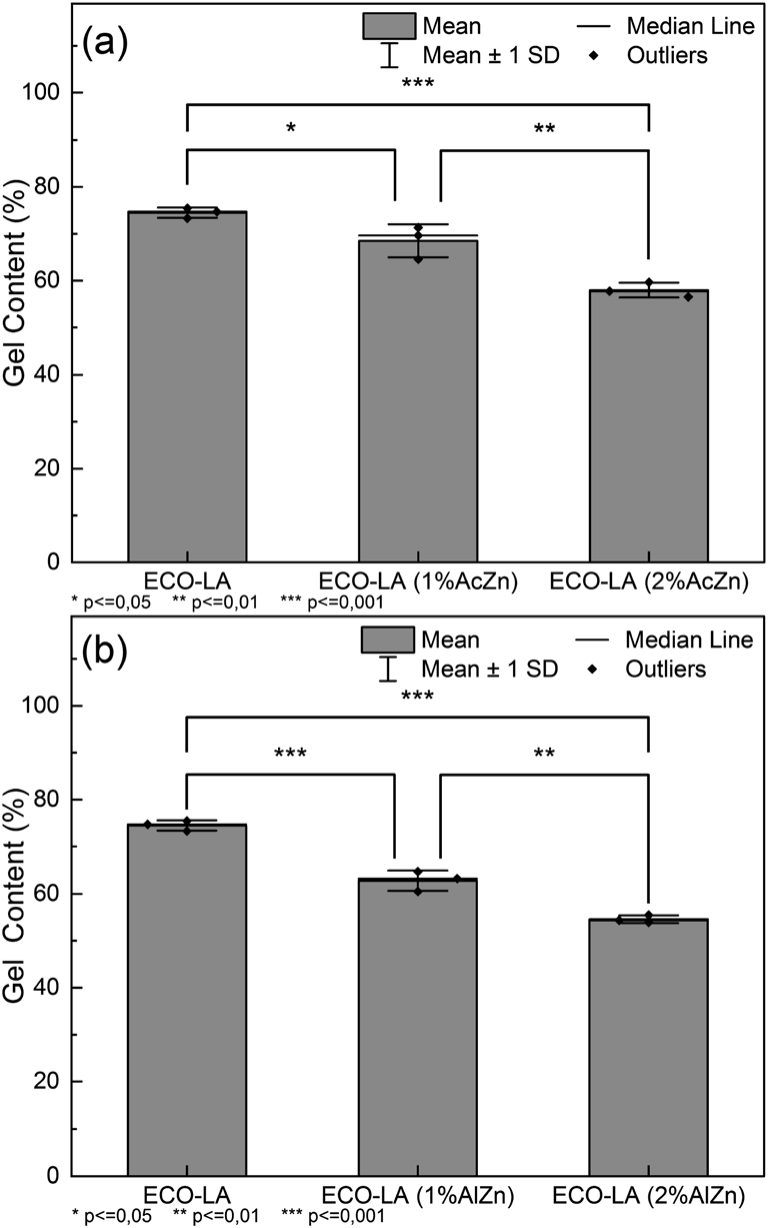
(a) Tukey test for samples cured with ZnAc at different concentrations, (b) Tukey test for samples cured with ZnAl at different concentrations.

An additional analysis using the Tukey Test helped to identify which concentrations showed a significant difference. Fig. 8a and b show the significant differences between the vitrimers containing different amounts of ZnAc and ZnAl.

For materials cured in the presence of ZnAc, a significant difference in the gel content between the material without a catalyst and those cured with 1 and 2 wt% of catalyst (*p* ≤ 0.05 and *p* ≤ 0.001) was observed. For the samples cured with ZnAl, the significance levels are higher in relation to the ZnAc catalysts (*p* ≤ 0.001 and *p* ≤ 0.001). The results suggest a statistical impact on the gel content by the catalysts.

### 3.4 Thermal stability

**Fig. 9:**
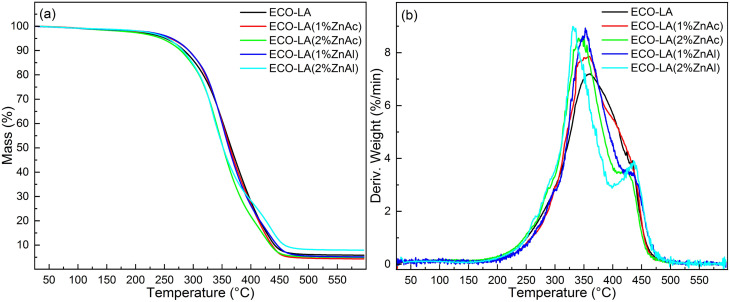
(a) Thermogravimetric analyses for ECO-LA vitrimers without a catalyst and with zinc-based catalysts at different concentrations (b) DTG curves.

TGA was used to characterize the thermal stability of the materials obtained in this work. Fig. 9 shows the thermogram obtained for materials cured without catalyst and in the presence of ZnAl and ZnAc catalysts at different concentrations.

Materials cured with 1 wt% of ZnAc and ZnAl showed a slight increase in the initial temperature degradation (*T*_onset_) in relation to the material prepared in absence of catalyst. The material made without catalyst had a *T*_onset_ of approximately 301 °C, and those with 1 wt% ZnAc and ZnAl had a temperature of 304 °C and 307 °C, respectively. Materials cured with 2 wt% of ZnAc and ZnAl showed a slight decrease in *T*_onset_ to 299 °C and 297 °C, respectively. The results demonstrate good thermal stability of these ECO-LA vitrimers. From the DTG curves, a tendency of a decrease in the temperature of maximum weigh loss rate (*T*_peak_) was observed. Materials cured with ZnAc presented a slight displacement of *T*_peak_ towards lower temperatures with increasing catalyst concentration. In materials obtained with ZnAl, the shift to lower temperatures was more pronounced. This phenomenon may be correlated with the density of crosslinks of the vitrimers.^52,60,61^

#### 3.4.1 Degradation kinetics

The kinetic of thermal decomposition of a polymeric system can be evaluated using the eqn (6), where *α* is the conversion, *W*_i_ is the mass at the initial temperature, *W*_T_ is the mass at a given temperature, and *W*_f_ is the mass at a final temperature.6
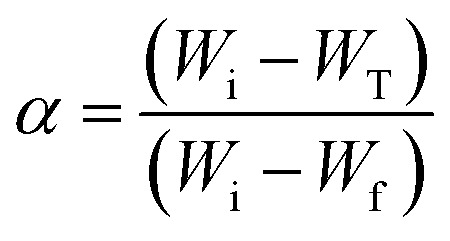


For kinetic studies, the activation energy and the pre-exponential factor can be calculated using different methods. These kinetic parameters can be calculated by model-free methods, where the Kissinger Akira Sunose (KAS) (eqn (7)) and Friedman methods (eqn (8)) are widely used.^62^7
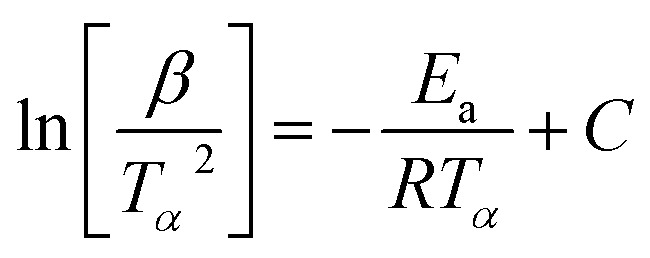
8
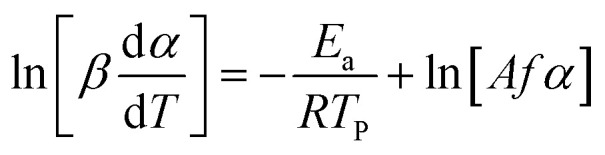


By the KAS method, the Activation Energy is obtained by taking the slope of the straight line plotting 
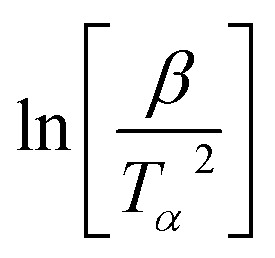
 as a function of 
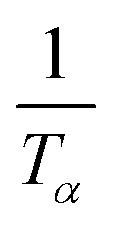
 (*T*_*α*_ is defined as the temperature at a given conversion, *α*) at a certain partial weight loss (*α*). Using the Friedman method, the activation energy as a function of conversion can be obtained byplotting 
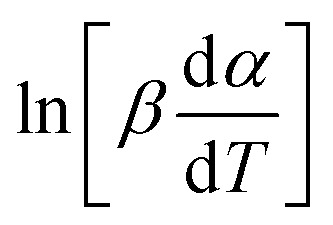
*vs.*
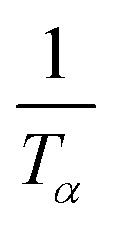
.

**Fig. 10:**
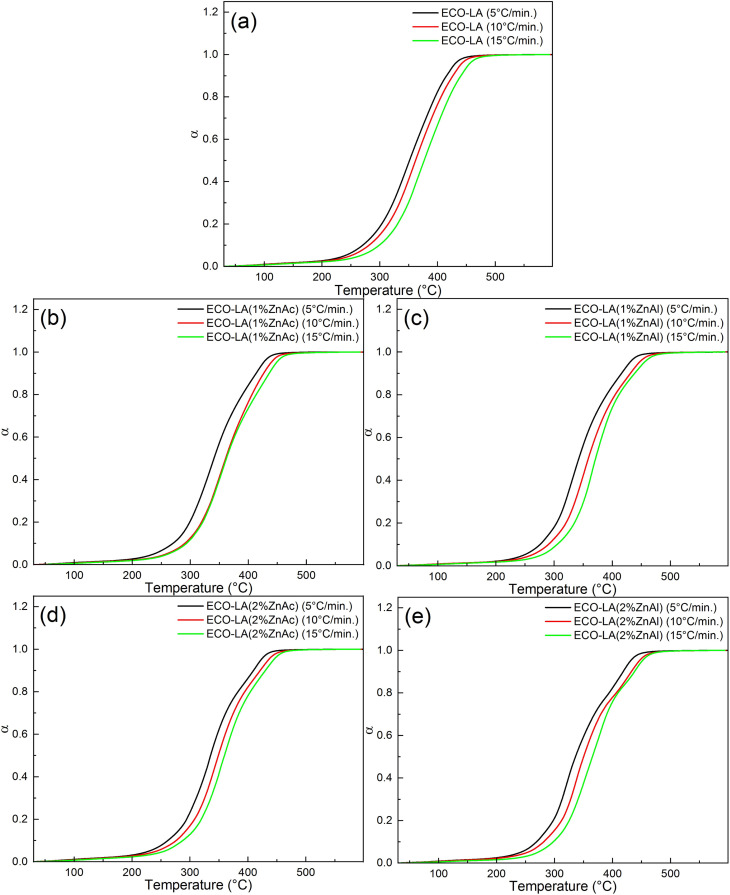
Conversion levels for different heating rates for (a) ECO-LA, (b) ECO-LA(1%ZnAc), (c) ECO-LA(1%ZnAl), (d) ECO-LA(2%ZnAc) and (e) ECO-LA(2%ZnAl).

The calculated conversion (*α*) as a function of temperature at different heating rates for all samples is shown in the Fig. 10. Note that the heating rate affects the degradation process. For higher heating rates, the degradation process is more accelerated, and the reaction is shifted to higher temperatures.^63^ This trend can be noticed for all samples, indicating that by placing different amounts of catalysts, the degradation reaction is dependent on the heating rates.

**Fig. 11:**
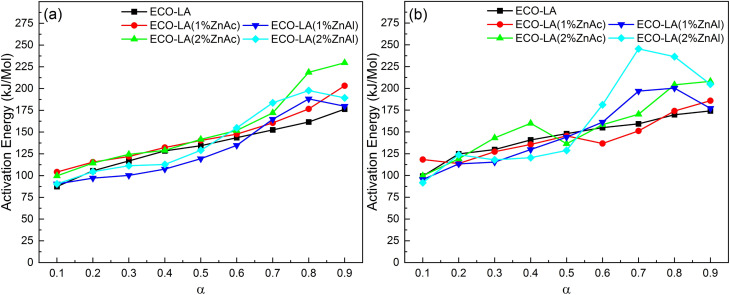
Distribution of activation energy as a function of conversion for samples without and with catalyst at different concentrations by the method (a) Kinssiger Akira Sunose and (b) Friedman.

Fig. 11 presents the activation energy *vs.* conversion fraction for the ECO-LA vitrimers. The activation energy is not constant as a function of the degree of conversion, suggesting that the decomposition takes place in several steps.^64^ The first step of degradation was observed in the range of *α* = 0.1–0.2. The low activation energies for material degradation in this *α* range may be associated with the degradation process of the fraction that did not polymerize during the curing process.^65^

In the range of *α* = 0.2–0.6, a minimum variation of the activation energy is observed. This behavior is associated with the degradation reaction of the material. It can be observed that the difference between samples is small, indicating that the addition of the catalyst does not interfere in this second stage of degradation.^66^

In the third step (*α* = 0.6–0.9), a pronounced increase in ac-tivation energies for materials containing the catalysts was observed. The increase in the catalyst concentration, increases in a more pronounced way the activation energy. This effect is associated with the formation of char which has a protective action on the polymer networks, suggesting that catalysts impact the final step of degradation.^67^

### 3.5 Thermal expansion behavior

Dilatometric analyzes were performed for these ECO-LA vitrimers at the temperature range of the rubbery state (25–250 °C). The topology freezing temperature represents the temperature where activation of bond exchange reactions occurs and the material reaches a viscosity higher than 10^12^ Pa s.^68,69^

For this transition temperature, a decrease in the dimension change rate caused by the softening of the material is expected. This dimensional change is associated with the bond exchange reactions present in the vitrimer. Thus, *T*_v_ can be determined as the temperature where the dimension variation reaches a maximum value, where onset softening occurs.^70^

**Fig. 12:**
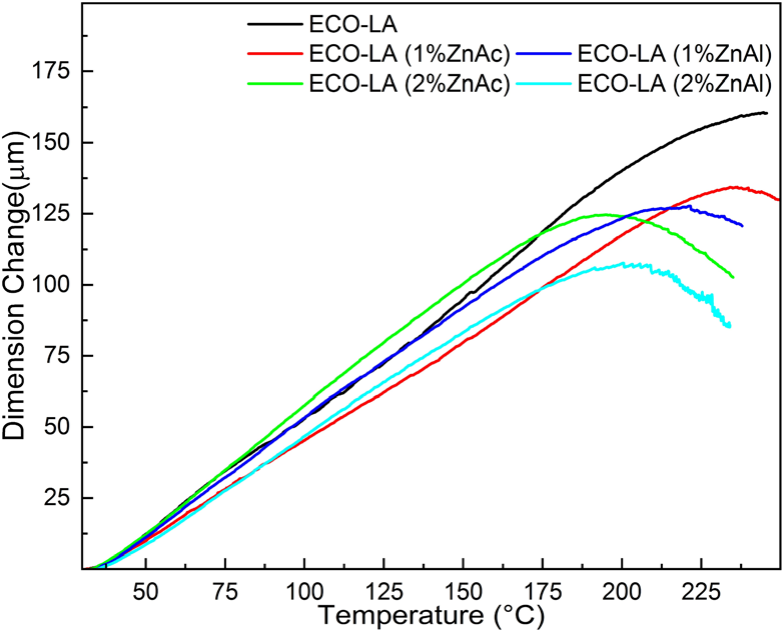
Thermal expansion curves for samples based on epoxidized canola oil cured at concentrations of 1 and 2 wt% ZnAc and ZnAl.

Fig. 12 shows the thermal expansion curves for the ECO-LA vitrimers cured without catalyst and in the presence of Zn catalysts at different concentrations. For the material cured without catalyst, a softening point was not observed in the temperature range under investigation, indicating that this material does not have vitrimeric characteristics, at least in this range of temperature below the onset degradation temperature for these materials. For the materials containing 1 wt% of ZnAc and ZnAl, a softening point can be observed at temperatures of 235 °C and 221 °C, respectively, indicating that these materials have vitrimeric characteristics. Materials cured with 2 wt% of ZnAc and ZnAl showed a decrease in *T*_v_ to 195 °C and 200 °C, respectively, demonstrating that the amount of catalyst influences this transition temperature.^70,71^

## 4 Conclusions

In this work we report an experimental investigation of the curing and thermal properties of biobased vitrimers of epoxidized canola oil and lactic acid using zinc acetate and ZnAl-layered double hydroxide as transesterification catalysts. For this system, the action of the catalyst as a reaction accelerator could be observed for both types of catalysts, but ZnAc presented better results in terms of cure. Spectroscopic analyses (FTIR) revealed that ring-opening polymerization of epoxy groups from the epoxidized oil generates polyhydroxyesters networks even in the absence of the catalysts. In general, these materials showed rubber-like properties with *T*_g_ ranging from −15 to −23 °C. Presence of the catalysts in the materials caused a reduction in the enthalpies of cure and interferes in the crosslinking density and gel content. Higher activation energies are achieved in the final step of thermal decomposition for vitrimers cured in presence of the catalysts. From thermal expansion experiments, materials containing the two catalysts showed a softening point at temperatures between 195–235 °C, associated with transesterification reactions and topology freezing temperature transition induced by the catalysts, which confirms the vitrimeric characteristics of the ECO-LA materials.

## Author contributions

Investigation and writing-original draft preparation, J. G. P. R.; conceptualization and writing-review and editing, M. L. D.; writing-review and editing, J. G. A. P.; investigation and editing, S. A. all authors have read and agreed to the published version of the manuscript.

## Conflicts of interest

There are no conflicts to declare.

## Supplementary Material
